# Development of Atypical Reading at Ages 5 to 9 Years and Processing of Speech Envelope Modulations in the Brain

**DOI:** 10.3389/fncom.2022.894578

**Published:** 2022-06-10

**Authors:** Raúl Granados Barbero, Pol Ghesquière, Jan Wouters

**Affiliations:** ^1^Research Group Experimental ORL, Department of Neurosciences, Katholieke University of Leuven, Leuven, Belgium; ^2^Parenting and Special Education Research Unit, Faculty of Psychology and Educational Sciences, Katholieke University of Leuven, Leuven, Belgium

**Keywords:** dyslexia, neural entrainment, auditory steady-state response (ASSR), source analysis, ICA, DSS, speech processing, EEG

## Abstract

Different studies have suggested that during speech processing readers with dyslexia present atypical levels of neural entrainment as well as atypical functional hemispherical asymmetries in comparison with typical readers. In this study, we evaluated these differences in children and the variation with age before and after starting with formal reading instruction. Synchronized neural auditory processing activity was quantified based on auditory steady-state responses (ASSRs) from EEG recordings. The stimulation was modulated at syllabic and phonemic fluctuation rates present in speech. We measured the brain activation patterns and the hemispherical asymmetries in children at three age points (5, 7, and 9 years old). Despite the well-known heterogeneity during developmental stages, especially in children and in dyslexia, we could extract meaningful common oscillatory patterns. The analyses included (1) the estimations of source localization, (2) hemispherical preferences using a laterality index, measures of neural entrainment, (3) signal-to-noise ratios (SNRs), and (4) connectivity using phase coherence measures. In this longitudinal study, we confirmed that the existence of atypical levels of neural entrainment and connectivity already exists at pre-reading stages. Overall, these measures reflected a lower ability of the dyslectic brain to synchronize with syllabic rate stimulation. In addition, our findings reinforced the hypothesis of a later maturation of the processing of beta rhythms in dyslexia. This investigation emphasizes the importance of longitudinal studies in dyslexia, especially in children, where neural oscillatory patterns as well as differences between typical and atypical developing children can vary in the span of a year.

## Introduction

Developmental dyslexia is a neurobiological and hereditary disorder characterized by severe and persistent difficulties with accurate and fluent word recognition and by poor spelling and decoding abilities (Eden et al., 2016), despite the normal intelligence and sufficient educational opportunity (Stoodley, 2016). Different studies have proven that individuals with dyslexia present deficits or atypical responses in the phonological components of language that causes problems to represent and manipulate the phonological structure of words at the syllable and/or phoneme level (Vellutino et al., 2004; Ziegler and Goswami, 2005; Vandermosten et al., 2020). However, the neural dysfunction behind these effects remains unknown. As it was stated in Share (2021) about the phonological deficit theory, phonemic awareness is only the “tip of the phonological iceberg” and that “deeper” spoken-language phonological impairments among people with dyslexia appear well before the onset of reading and even at birth (Guttorm et al., 2005).

Atypical development has been observed for persons with dyslexia. One of the experimental techniques to measure brain electrical responses is the electroencephalography (EEG). EEG can measure the electrical impulses of the brain (responses) at the scalp level. EEG can allow us to measure the neural entrainment of the brain to sensory stimulation.

Neural entrainment or neural synchronization is an important characteristic of interactions between brain rhythms or between brain activity and sensory stimulation. It refers to the coupling of two independent oscillatory systems in the brain or to the coupling of brain activity to the oscillatory properties of sensory stimulation in such a way that their periods of oscillation at specific frequencies become related by virtue of phase alignment (Cummins, 2009). A higher entrainment or synchronization of the brain to sensory oscillatory stimulation usually means higher brain responses at the frequencies of stimulation. Thus, we used the broader definition of neural entrainment or synchronization in the broad sense presented in Obleser et al. (2008).

A number of studies have tested this hypothesis in different age populations, with different methodologies and different oscillatory ranges (McAnally and Stein, 1997; Menell et al., 1999; Lehongre et al., 2011, 2013; Hämäläinen et al., 2012; Poelmans et al., 2012; Vanvooren et al., 2014; Lizarazu et al., 2015; De Vos et al., 2017a; Granados Barbero et al., 2021b).

These previous findings confirmed that a dyslectic brain could present atypical synchronization behavior in beta and low gamma rhythms. Despite being suggested that dyslexia should be a theta-related issue (Goswami, 2011), no consensus was found in the results regarding the differences during theta stimulation.

However, we cannot say which of these atypical neural effects observed in these previous studies are causally related to the development of dyslexia. An important shortcoming of these studies is that the observed neural deviances might reflect the resulting effects of the reading difficulties. Longitudinal studies in dyslexia aim to reduce the impact of those limitations, that is why, they are now considered essential to improve our knowledge about dyslexia (Goswami, 2015).

The main objective of this study was to evaluate the relationship of the neural synchronization with speech envelope modulation rates during reading development. We embarked upon a longitudinal study to analyse the neural entrainment during auditory stimulation in typically developing children and children with dyslexia at the ages of 5, 7, and 9 years old.

Part of these data were used in the study by De Vos et al. (2017a). However, we used an extended source analysis methodology first developed in Granados Barbero et al. (2021a). We hypothesized that this approach will provide us with essential information about how neural processing during speech related modulations evolves with age as well as its impact in children with dyslexia.

We selected a brain source analysis to avoid defining regions of interest at the scalp level; furthermore, with our method selection, we could analyse our outcome measures the most prominent brain generators in the same group with no prior assumptions of our data. It is true that we are neglecting the role that inter-subject variability plays in phase difference; however, our main goal was to extract the information maximally shared by all the subjects in a group. Source analyses have been used widely in the literature for their ability to provide with physiologically plausible results with no prior assumption in the location of the sources. Almost, no assumption is also taken regarding the relationship between the reconstructed signals. This approach offers the flexibility to look for specific signals in the brain without taking any important prior assumptions.

It has been suggested the use of multivariate regression models. However, we decided to use a method with the input parameters well defined (EEG channel data). Multivariate methodology bases its use in machine learning algorithms that can lead to excellent predictions, but sometimes to not very useful interpretations in terms of brain function (Hebart and Baker, 2018). To avoid this, normally, big parts of data are used for training, regions of interest are defined within the brain, or other constrictions are applied to the reconstructed data, such as predefining the number of sources. In addition, common multiple regression models used in EEG analyses aim to extract signal predictors based on the spectral properties of a complex signal. They need the temporal and the spectral (frequency) information of the signal as an input. They are mostly used with event-related potentials, which contain information widespread in the frequency domain. Since we aimed to stimulate at certain modulation frequencies to resemble specific parts of human speech, we do not have such a rich frequency spectrum. Therefore, we aimed to have a methodology that could work with oscillatory signals happening in a narrow frequency window as well as having the minimum number of assumptions regarding our data.

The analyses performed in the EEG recorded brain responses included the following: (1) source localization, (2) the estimation of the hemispherical preference of the ASSR sources using a laterality index and the measure of neural entrainment combining the results provided by the (3) SNRs and the (4) phase coherence at the modulation frequencies. The SNR and the phase coherence provided the information of the main ASSR generators' oscillatory activity. The SNR measured the evoked responses compared to the background noise, and phase coherence quantified the connectivity by measuring the phase difference variability of the main ASSR brain generators.

## Materials and Methods

### Data Acquisition

#### Participants

The original sample consisted of 87 children with bilateral normal hearing, normal non-verbal IQ, and no history of brain injury or neurological disorders (for a detailed description of the original sample, refer to Vanvooren et al., 2014). A total of 14 children did not continue their participation in the study, one child was excluded following a diagnosis of general learning difficulties, and EEG data of four children were considered unusable because of overall too high noise levels. Consequently, we were able to retain a longitudinal sample of 68 children for the data presented here. The division in the two groups for the study: readers with and without dyslexia was done retrospectively. Participants were classified as typical readers (TR) or readers with dyslexia (DR) based on their history of reading problems and their current reading performance as well as their spelling history and their current spelling performance. More specifically, children diagnosed with dyslexia demonstrated (1) severe and persistent reading problems, implemented as a score below the 10th percentile on the same standardized word reading test (Brus and Voeten, 1973) or pseudoword reading (Van den Bos et al., 1994) at each measurement point, or (2) severe and persistent spelling problems, implemented as a score below the 10th percentile on a standardized spelling test (Dudal, 1997) at each measurement point. All participants were monolingual Dutch speakers, without a history of brain damage, permanent hearing loss, or visual problems. Additionally, participants were required to have adequate nonverbal intelligence, defined by an IQ score of > 85 on the Raven Colored Progressive Matrices (Raven and Court, 1996) measured at kindergarten age. More details about this classification as well as the reading and spelling performance can be found in De Vos et al. (2017b). Since the number of readers with dyslexia was much smaller than the typical readers, we selected the best readers from the control group to have a matched number of participants in each group based on the word recognition test (Brus and Voeten, 1973). We selected the best readers because dyslexia is mostly defined as a reading impairment. Therefore, for the diagnosis of dyslexia, children fulfilling the second criterion (severe and persistent spelling problems) were additionally required to demonstrate reading scores below the 25th percentile at each measurement point. Some of the poor readers not diagnosed with dyslexia belonged to this percentile, we did not want to have overlapped samples in reading performance. We were aware that there might be better choices to separate the effects inherent to dyslexia from effects derived simply by poor reading or spelling performance. However, since dyslexia has been widely defined as a reading impairment, the diagnosis of children is mostly based on reading performance. In addition, a more appropriate selection of the control group would only try to minimize the difference between observing effects caused by dyslexia or by reading performance. Thus, based on our selection method, the total number of participants was 21 for each group. EEG data were recorded from the same group of children in different stages: the first stage corresponded to a pre-reading stage where the participants' mean age was 5 years; in the second stage, the children were in a beginning reading state and were 7 years of age on average; the third stage corresponded to an advanced reading state where the mean age of the group was 9 years.

#### Auditory Steady-State Responses

Auditory steady-state responses (ASSRs) are phase-locked electrophysiological responses from the brain, evoked by a periodically varying continuous auditory signal (Picton et al., 2001; Rance, 2008). ASSRs allow an objective investigation of auditory temporal processing in the brain and brainstem by adjusting the stimulation parameters to match important speech-related components (Lins and Picton, 1995).

The stimuli used to evoke these responses consisted of amplitude modulated speech-weighted noise. The carrier noise was adopted from the *Leuven Intelligibility Sentence Test* (LIST) (Van Wieringen and Wouters, 2008). This carrier noise represents the long-term average speech spectrum of all sentences of the female speaker with LIST readings. The speech-weighted carrier noise was 100% amplitude modulated at approximately 4 and 20 Hz rounded to the epoch frequency of 1/1.024 Hz (exact frequencies were 3.91 and 19.53Hz), representing syllable- and phoneme-rate modulations, respectively. With this stimulation, we aimed to measure synchronization of neural oscillations in the auditory cortex. All stimuli were presented at 70 dB SPL using the Etymotic Research ER-3A insert earphones monaurally to the right ear. This was done for a matter of time since the participants were children and to avoid the simultaneous activation of both auditory pathways.

#### Recording System

Electroencephalography data were recorded with the BioSemi ActiveTwo system using 64 active Ag/AgCl electrodes mounted in head caps according to the 10-10 electrode system. Electrode offsets were kept below 30 mV. All recordings were administered in a double-walled soundproof booth with a Faraday cage. Participants were asked to lie down on a bed while watching a soundless film to warrant the same level of alertness and attention throughout the EEG measurement (Poelmans et al., 2012; Vanvooren et al., 2014; Goossens et al., 2016). Each measurement consisted of a 10-min EEG recording and they were presented randomly to avoid any influence of the stimulation order.

### Pre-processing

All data were pre-processed using MATLAB^®^. First, EEG signals were filtered using a zero-phase high-pass filter with a cutoff frequency of 2 Hz and a slope of 12 dB/Octave. The filtered signal was segmented into different epochs with a length of 1.024 s. From these segmented epochs, we applied the following steps: the mean peak-to-peak (PtoP) amplitude was calculated for each channel separately. Any channel with a mean PtoP four times greater than the median PtoP of all the channels was rejected, and any subject with rejected channels was discarded. The aim was to obtain 128 epochs with the lowest amount of artifacts in all the channels. To do that, we considered the 128 epochs showing the lowest absolute amplitude. If one of these selected epochs had reached a maximum threshold of 120 μV the subject was discarded. As it was mentioned in the participants Section Discussion, participants were discarded due to the high noise levels. The accepted 128 epochs per subject were concatenated along the time dimension, resulting in a total array of 2,560 epochs per EEG electrode for each group of children. After the epoch-based artifact rejection, all the electrodes were referenced to the Cz electrode. This reference was used over the average reference based on the previous studies that stated that Cz reference lowers the noise estimates around the stimulation frequency reducing the detection threshold for ASSRs (Van der Reijden et al., 2005; Van Dun et al., 2009; Poelmans et al., 2012).

### Source Activity Reconstruction

To extract ASSR brain source activity, we combined independent component analysis (ICA) and denoising source separation (DSS), as described in the study by Granados Barbero et al. (2021a). We aimed to extract the most reproducible ASSR across trials and to identify patterns within the whole group for each age. Time-concatenated EEG data were decomposed by ICA into an array of maximally independent components (Onton, 2009). The brain source localization and projection weights were assumed to be spatially stationary for the duration of the experiment (Onton et al., 2006). The Infomax algorithm was utilized in FieldTrip (Oostenveld et al., 2011) due to its fast and reliable performance (Bell and Sejnowski, 1995). Since we applied ICA to temporally concatenated data, we were performing what is known as group-ICA (Delorme and Makeig, 2004). For group-ICA, we applied ICA to temporally concatenated data from all the subjects in each group and for each age separately. The number of output components equalled the number of input channels, which were 63 per stimulation condition. After computing ICA, a one-sample Hotelling *t*-squared test was performed, refer to section Detecting Significant ASSR. Components that did not show significant ASSR were rejected.

The non-rejected ICA components were inputted into the DSS algorithm. DSS performs a separation of the data into desirable components (signal) and undesirable components (noise), assuming linearity of the coefficients and of the DSS components (Särelä and Valpola, 2005).

Denoising source separation can use a bias function to enhance its sensitivity toward phase-locked ASSR. This bias function was chosen to be the *proportion of epoch-averaged (evoked) activity* (de Cheveigne and Simon, 2008). This bias function represented the total-power reproducibility across trials. It allows to rank the DSS components according to the most consistent activity along trials. Since the stimulation during the experiment was periodical and consistent across trials, we expected the activity of the main ASSR phase-locked generator to be ranked first.

After computing the DSS algorithm, we obtained an array of components sorted by decreasing total-power reproducibility across trials. To determine whether these DSS components were carrying ASSR, a one-sample Hotelling *t*-squared test was computed, refer to the section Detecting Significant ASSR.

**Figure 1:**
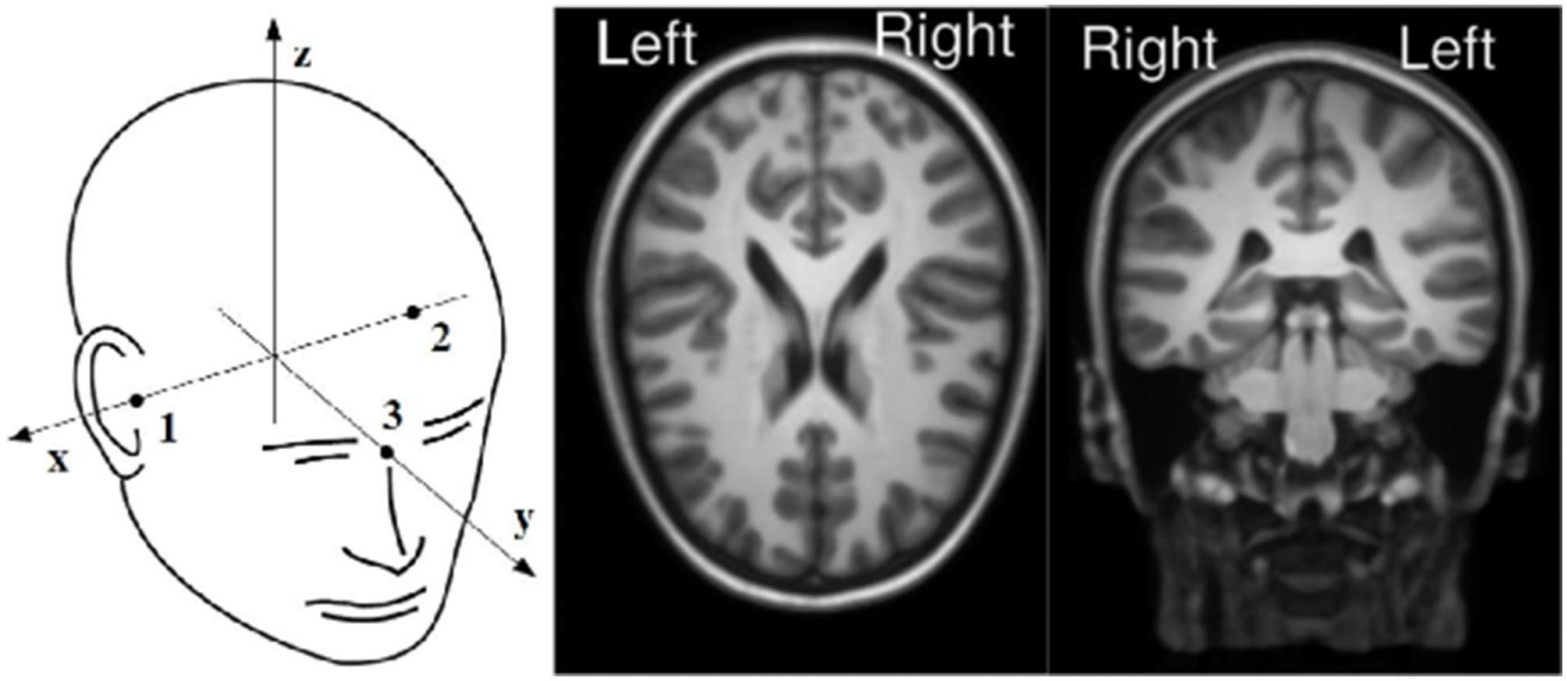
**On left panel**: Neuromag coordinate system. **On right panel**: MRI orientations used for the visualization of the dipoles fitted.

The projection weights of the components obtained through DSS and ICA analyses were fitted to equivalent current dipoles using the Neuromag coordinates, refer to Figure 1. A total of two symmetrical dipoles along the x-axis were the default configuration as long as the distance between them was more than 20 mm, the one dipole configuration was selected otherwise. This dipole fitting procedure was performed using a boundary element method (BEM) as a head model (Hämäläinen and Sarvas, 1989; Meijs et al., 1989). We tried to create age-related (5, 7, and 9 years old) BEM head models from averaged MRIs. However, the tissues were so thin, especially in the parietal areas, that they created distortions in the surface boundaries. These distortions led to the creation of unusable BEM head models for the three ages involved in the experiment. The limitations encountered while trying to create an age-related model were solved using the template based on older subjects. This BEM conductivity model was created in FieldTrip from 14-year-old adolescents averaged MRI images (Sanchez et al., 2012; Richards et al., 2016). The BEM model described the electrical properties of the scalp, skull, and brain and the conductivity values chosen for these tissues, which were 0.3300, 0.0042, and 0.3300 S/m, respectively (Gabriel et al., 1996).

### Outcome Measures

In this study, our main outcome measures were response strength (SNR), connectivity (phase coherence), and lateralisation (laterality index).

#### Signal-to-Noise Ratio

The measured relative response amplitudes, obtained from the square root of the signal power at the frequency of stimulation, and the signal-to-noise ratios (SNRs) were calculated from the DSS components carrying significant ASSR. We obtained these responses and SNRs from the concatenated data for both stimulation conditions, in both groups and for all ages. The response amplitudes were obtained by taking the square root of the signal power. The signal power, *P*_*S*_, was calculated from the Hotelling *t*-squared statistic, and it represents the squared amplitude of the response mean across all the data trials at the bin of the modulation frequency.

The noise floor amplitude, *P*_*N*_, required to compute the SNR, was also estimated from the Hotelling t-squared statistic and shows the standard error of the amplitude responses at the modulation frequency *mf* across *n* epochs, being: $\sigma \left(R_{mf}\right)/\sqrt{n}$. Based on the definition of power response and noise amplitude, the SNR may be defined as the ratio between the power of the response signal and the power of the EEG noise, Equation 1.


(1) $$SNR=\frac{P_{S}}{P_{N}}=\frac{\|mean(R_{mf}){\|}^{2}}{{\left(\frac{\sigma (R_{mf})}{\sqrt{n}}\right)}^{2}}$$

where *R*_*mf*_ is a complex vector containing the responses at the modulation frequency of all epochs, σ is the standard deviation, and *n* is the number of epochs.

#### Phase Coherence

Phase coherence represents the inter-trial variability of the phase difference between two signals for a specific frequency, and it can be interpreted as a measure for connectivity (Picton et al., 2001).

We measured the phase coherence between the first two DSS components. The first and the second DSS components carry the highest SNR of all the components as well as the highest reproducibility power ratios. We did not include following DSS components because we cannot guarantee the presence of a dipolar pattern. From the third and following DSS component, the field spread may represent or contain a superposition of neural activity as well as noise sources (Granados Barbero et al., 2021a). This means that those two components gave us an overall model or estimation of most of the ASSR activity (in terms of energy/amplitude) triggered by our auditory stimulation. Through the phase coherence, we obtained an estimation of how the two main ASSR generators oscillate with each other. A higher capability for phase locking means a higher level of synchrony, or in other words, it means a stable phase difference between the two main ASSR generators. The phase coherence showed the synchrony that can be found between two signals. This was made by estimating how stable the difference between the phase of two signals is.

In summary, the phase coherence measured the neural interaction of the activity generated during this stimulation. Higher values meant a higher synchronization or joint effort toward “decoding” or processing the stimulus. The phase coherence was computed after averaging segments of data into 32 epochs, as suggested in the study of Picton et al. (2001). The phase of each epoch was obtained from the complex representation of the neural response in the frequency domain. Phase coherence was calculated according to Equation (2) (Picton et al., 2001).


(2) $$PhCoh=\frac{1}{n}\sqrt{{\left(\sum_{i=1}^{n}cos\;\theta_{i}\right)}^{2}+{\left(\sum_{i=1}^{n}sin\;\theta_{i}\right)}^{2}}$$

where θ(*f, i*) represents the phase difference between two signals, in this case, the selected signals would be the first and the second computed DSS components, and this can be expressed as follows: ϕ_1_(*f, i*) − ϕ_2_(*f, i*). For the final result, the difference will be averaged across *n* epochs.

#### Laterality Index

To estimate the lateralisation of the extracted sources, we computed the laterality index (LI). The neural origin for the DSS components was estimated by the dipole fitting procedure. This neural origin could be modeled by one or two symmetrical dipoles, as it was mentioned before while describing the dipole fitting procedure. For components whose origin was modeled by two symmetrical dipoles, we defined the LI based on the dipole magnitudes as it is shown in Equation (3). For components modeled by only one dipole, we calculated the LI based on the reconstructed responses and the noise level of the extracted component. If the dipole was in the right hemisphere (*x*_*dip*_ > 0), the noise floor was used to estimate the neural response of the left hemisphere, refer to Equation (4). If the dipole was in the left hemisphere (*x*_*dip*_ < 0), the noise floor was used to estimate the neural response of the right hemisphere, refer to Equation 5. Either with one or two dipoles, an LI of +1 represents a response completely lateralised to the right hemisphere and an LI of −1 represents a response completely lateralised to the left hemisphere.


(3) $$LI=\frac{mag_{right}-mag_{left}}{mag_{right}+mag_{left}}$$

(4) $$if\;x_{dip}>0\;then\;LI=\frac{\sqrt{P_{S+N}}-\sqrt{P_{N}}}{\sqrt{P_{S+N}}+\sqrt{P_{N}}}$$

(5) $$if\;x_{dip}<0\;then\;LI=\frac{\sqrt{P_{N}}-\sqrt{P_{S+N}}}{\sqrt{P_{N}}+\sqrt{P_{S+N}}}$$

### Statistical Analyses

#### Detecting Significant ASSR

To determine whether the ICA or the DSS components were carrying ASSR or not, the one-sample Hotelling *t*-squared test was performed in the frequency spectrum of the components (Hotelling, 1931). These spectra were obtained by means of a discrete Fourier transformaation. The statistical test compared the complex responses at the modulation frequency bin across *n* different epochs.

Obtained components were considered to have ASSR activity when the Hotelling t-squared test showed a significant difference (α ≥ 0.05) between the squared mean of the response, numerator of Equation (1), and the squared standard error of the *R*_*mf*_ distribution, denominator of Equation (1) (Hofmann and Wouters, 2012).

#### Error Estimation for Group Analysis

The Quenouille and Tukey's jackknife approach was utilized to estimate bias and variance (σ_*JK*_) for the group analysis (Miller, 1974). The standard deviation of the mean value μ, which is the value including all *M* subjects, was estimated from the jackknife estimate of variance, refer to Equation 6. This jackknife statistic estimation is assumed to have a normal distribution (Efron and Stein, 1981).


(6) $$\begin{array}{r} \sigma_{JK}=\sqrt{\frac{M-1}{M}\overset{M}{\underset{m=1}{\sum }}\left[\zeta_{m}-\zeta_{all}\right]} \end{array}$$

where ζ_*all*_ represents the data of all subjects, ζ_*m*_ represents the data for one subject, and *M* is the total number of subjects.

#### Two Tailed *t*-Test

Based on the mean and standard deviations obtained using the jackknife method for the outcome measures, we calculated how significant the differences were between the control group (TR) and the group with dyslexia (DR) for the three age points using a paired *t*-test. We used Bonferroni–Holm correction (Holm, 1979) every time a multiple comparison was performed.

## Results

### Source Localization

**Figure 2:**
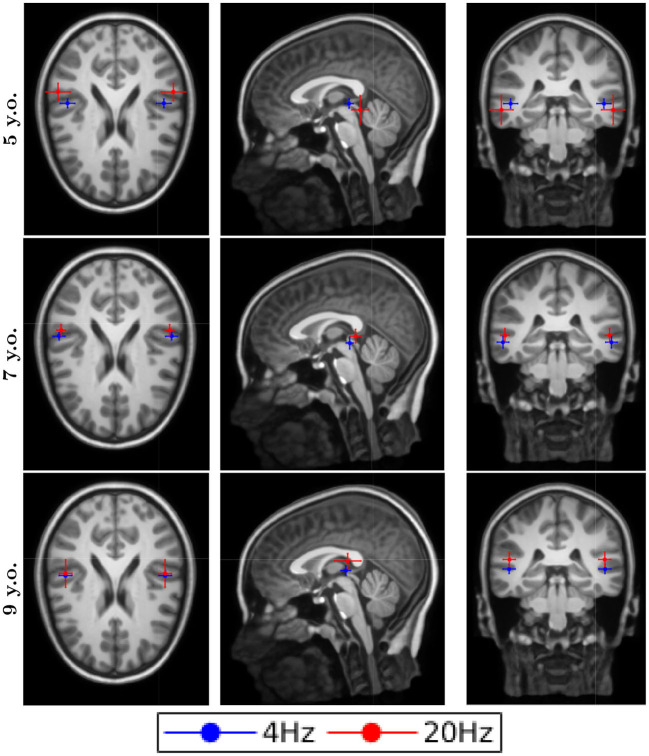
Dipole locations for the typical readers along different ages.

**Figure 3:**
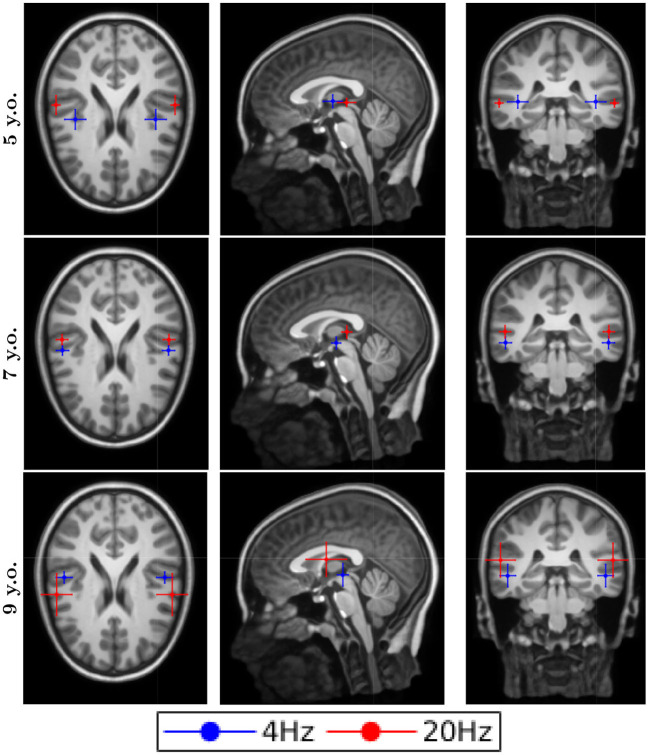
Dipole locations for the readers with dyslexia along different ages. They represent the asterisks that are shown in the figure, which they represent significance levels.

The dipole locations for the first DSS component are shown in Figure 2 for the typical reader group and in Figure 3 for the readers with dyslexia. All the responses were processed in both hemispheres for all conditions, both groups and all ages. The averaged location for all the conditions was in the temporal gyrus. Although the error bars showed the subject dispersion around the mean, the low spatial resolution of EEG in addition with the lack of individual head models produced a decrease of the source localization accuracy that was not reflected in the length of these error bars. Thus, we cannot assess whether there are differences in the source localization for the two groups under study due to their proximity and the spatial resolution issues.

### Response SNR

**Figure 4:**
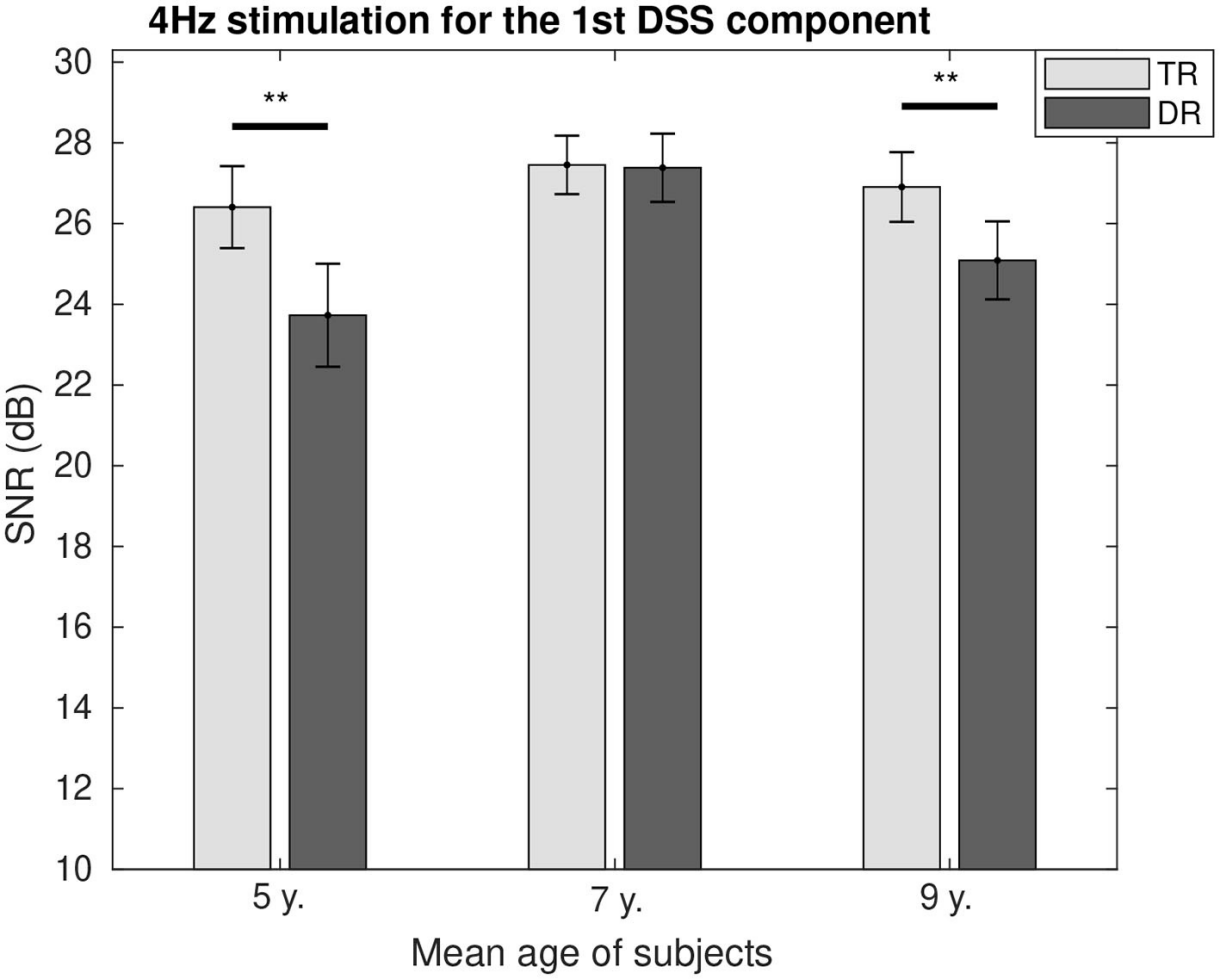
Group comparison of the SNR for the first DSS component at 4 Hz. Significant differences below a 0.05 threshold are marked with an * and thresholds below 0.01 are marked with ** for all figures. All the *p*-values were corrected using the Bonferroni–Holm approach. They represent the asterisks that are shown in the figure, which they represent significance levels.

**Figure 5:**
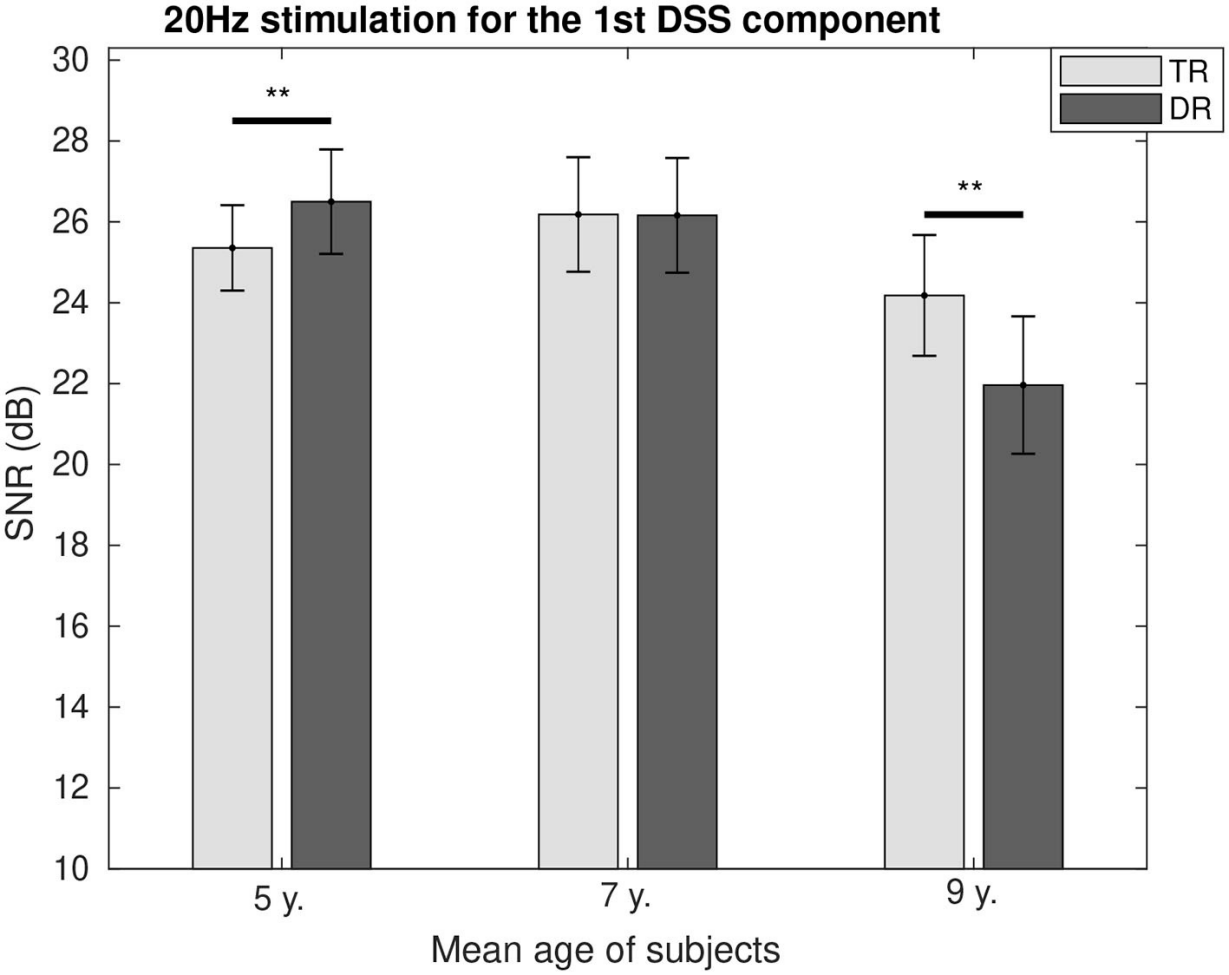
Group comparison of the SNR for the first DSS component at 20 Hz. Significant differences below a 0.05 threshold are marked with an * and thresholds below 0.01 are marked with ** for all figures. All the *p*-values were corrected using the Bonferroni–Holm approach.

The SNR comparison between the two groups as a function of age is represented for 4 Hz in Figure 4 and for 20 Hz in Figure 5. It can be seen how at the age of 7, we could not find the differences between the two groups for both modulation frequencies. While for 4 Hz, the SNR was lower for the group with dyslexia at the age of 5 (*p* < 0.01) and 9 (*p* < 0.1); and for 20 Hz, the SNR fluctuated from a higher value for the group with dyslexia at the age of 5 (*p* < 0.1) to a lower SNR for the group with dyslexia at the age of 9 years (*p* < 0.01).

### Phase Coherence

**Figure 6:**
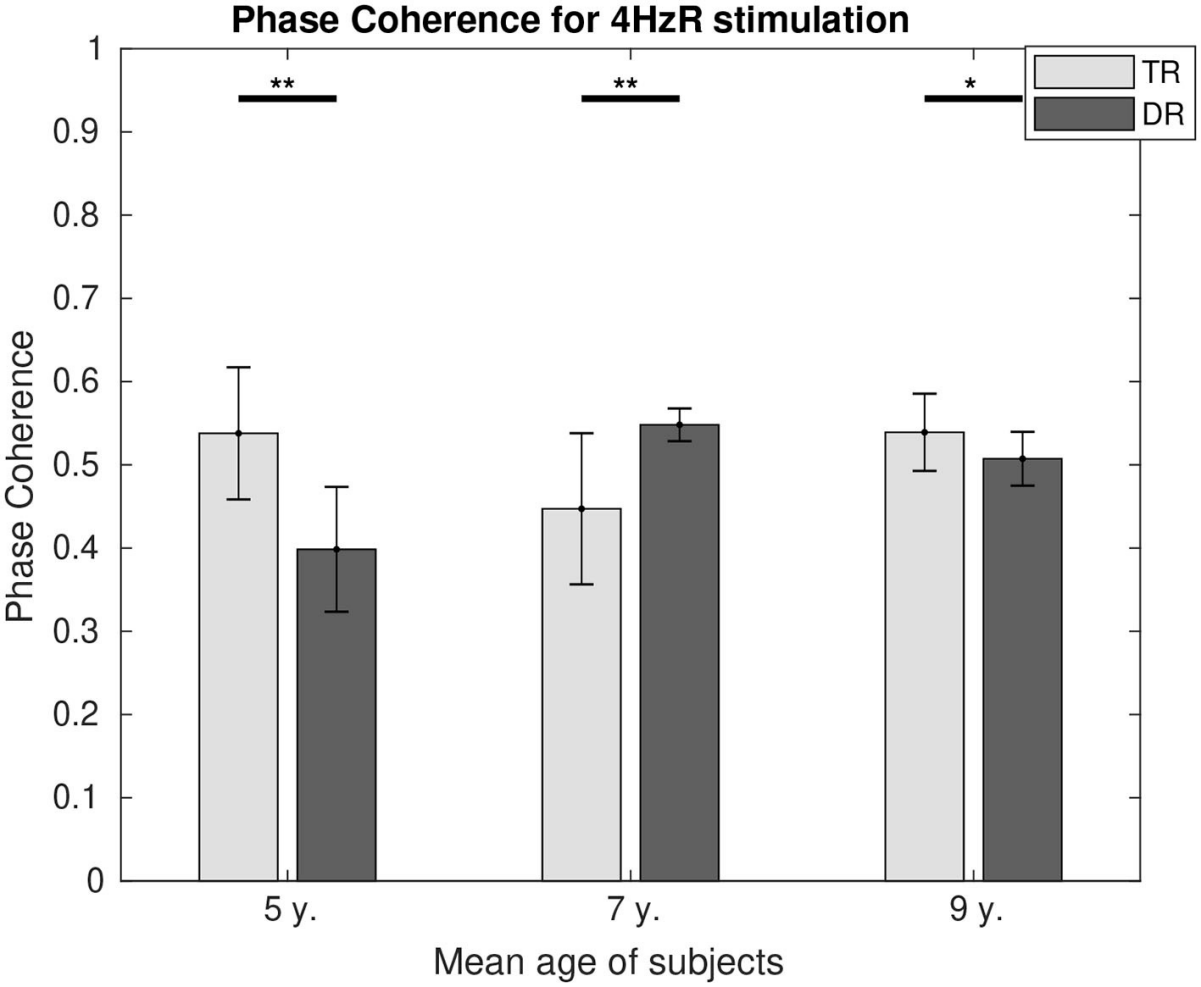
Comparison of the phase coherence for the two first DSS components at 4 Hz for both groups. Significant differences below a 0.05 threshold are marked with an * and thresholds below 0.01 are marked with ** for all figures. All the *p*-values were corrected using the Bonferroni–Holm approach. They represent the asterisks that are shown in the figure, which they represent significance levels.

**Figure 7:**
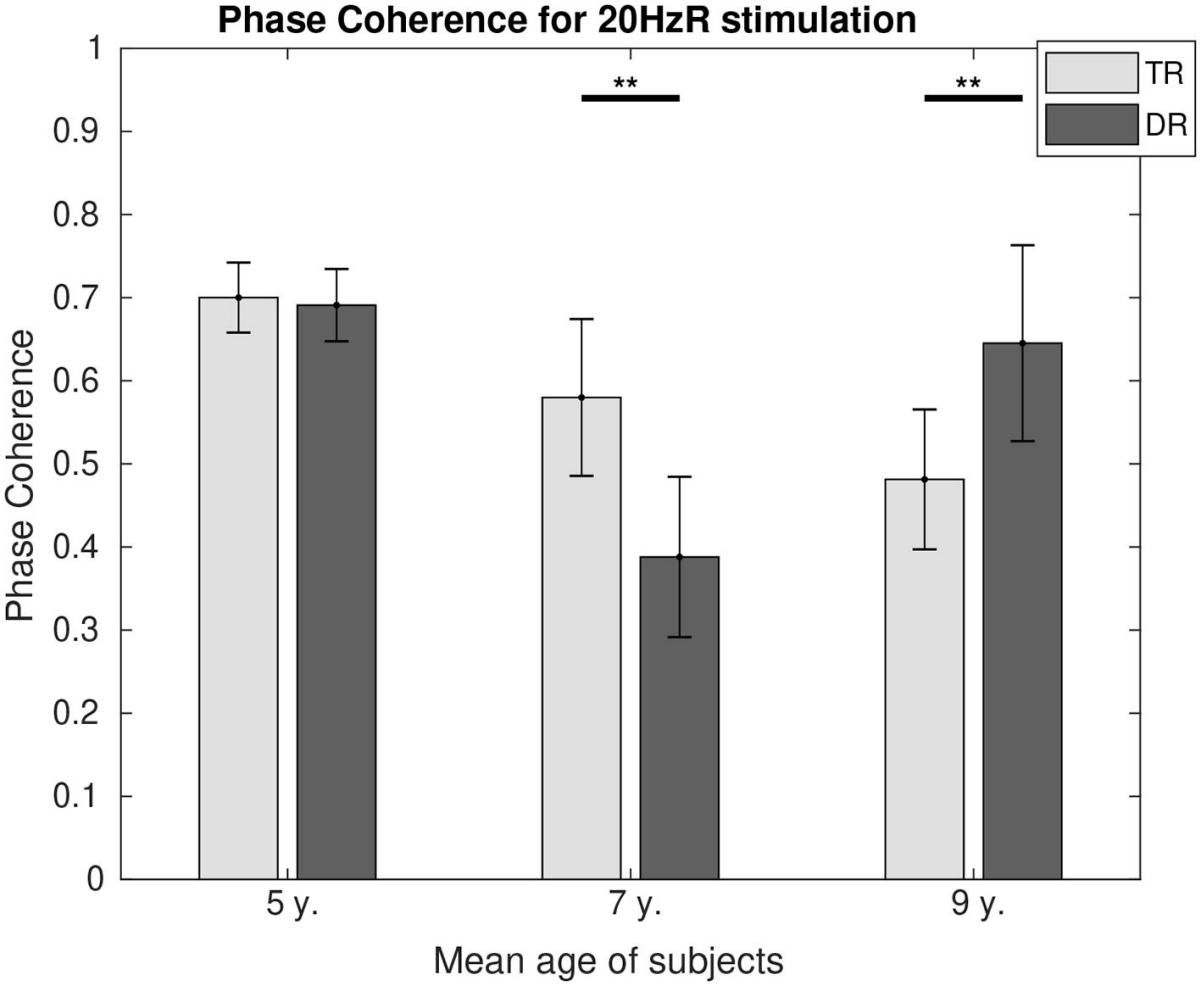
Comparison of the phase coherence for the two first DSS components at 20 Hz. Significant differences below a 0.05 threshold are marked with an * and thresholds below 0.01 are marked with ** for all figures. All the *p*-values were corrected using the Bonferroni–Holm approach.

Phase coherence values can be found in Figure 6 for 4 Hz and in Figure 7 for 20 Hz. In both figures, it is represented how the differences in phase synchrony evolved with age between the typically developing group and the group with dyslexia. For 4 Hz, the typical readers presented a higher connectivity at the age of 5 (*p* < 0.01) and 9 (*p* < 0.05), whereas at the age of 7 (*p* < 0.01), the group with dyslexia showed a higher phase coherence. For 20 Hz, there were no differences at the age of 5, and at the age of 7 (*p* < 0.01), the typical readers presented higher connectivity values, whereas at the age of 9 (*p* < 0.05), the group with dyslexia presented higher phase coherence values.

### Laterality Index

**Figure 8:**
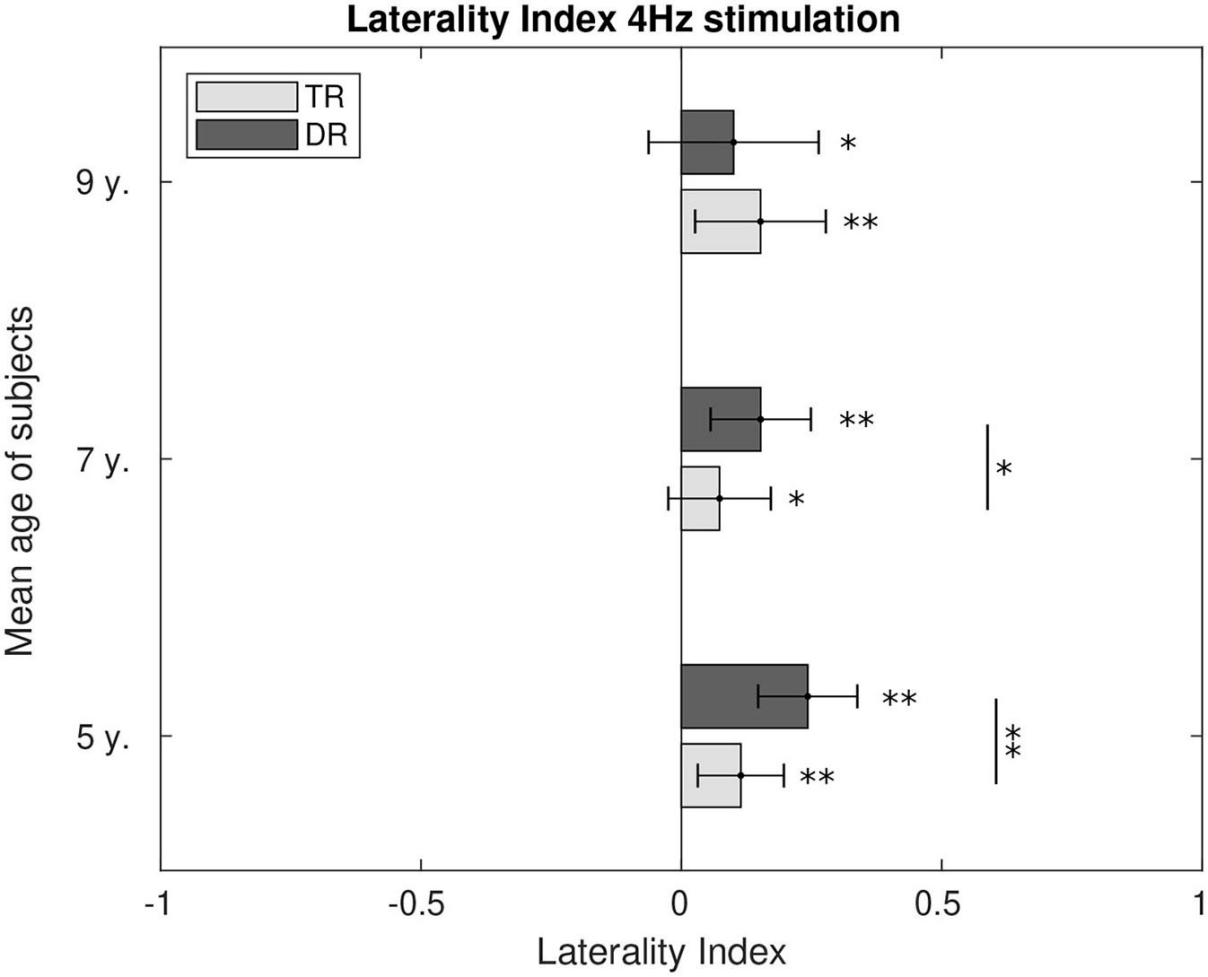
Comparison of the laterality index of the first DSS component at 4 Hz. LI values significantly different from zero were calculated and are marked in the figure as well as the differences between the two groups. *p*-Values below 0.05 are marked with an * and *p*-values below 0.01 are marked with ** for all figures. All the *p*-values were corrected using the Bonferroni–Holm approach.

**Figure 9:**
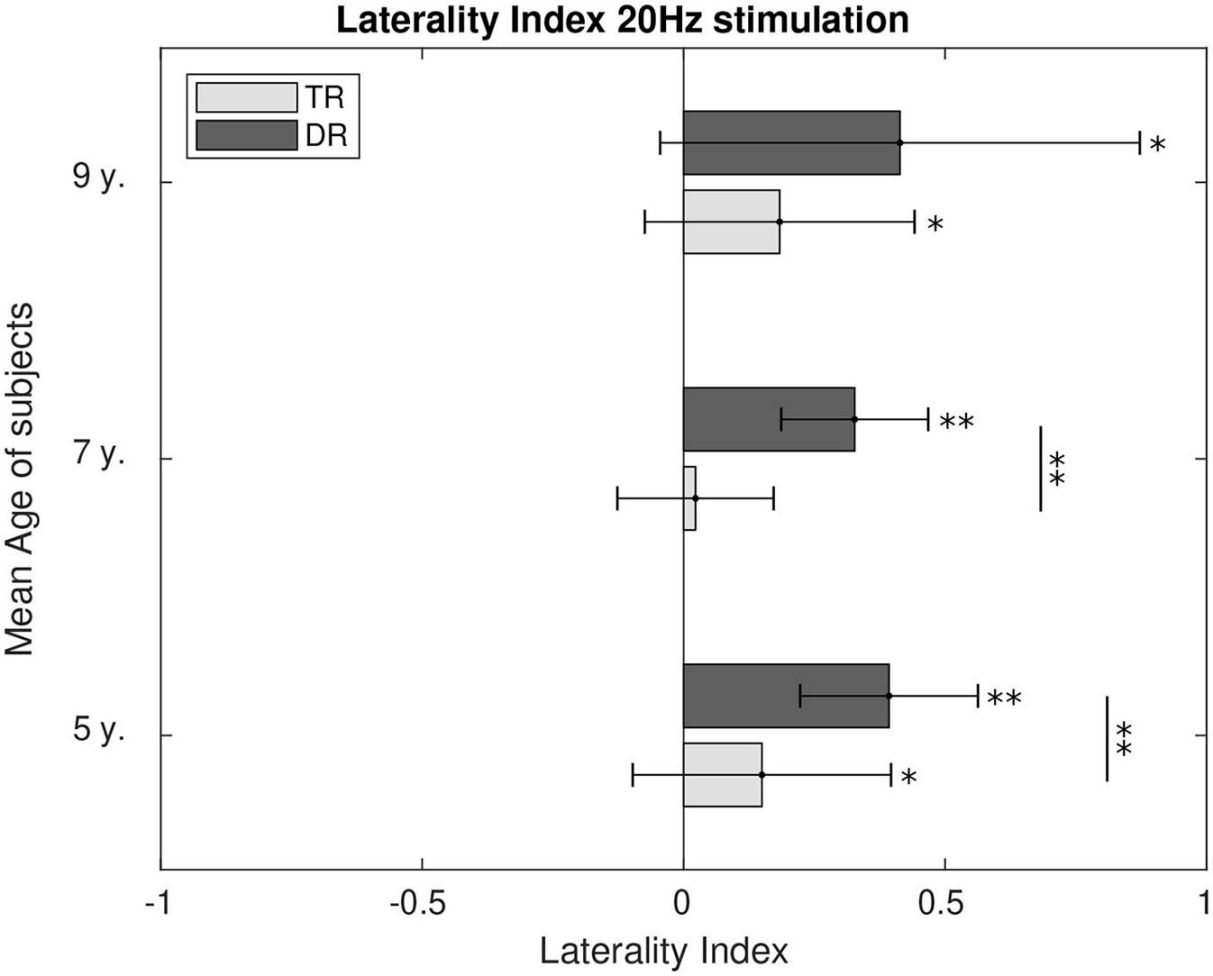
Comparison of the laterality index of the first DSS component at 20 Hz. LI values significantly different from zero were calculated and are marked in the figure as well as the differences between the two groups. *p*-Values below 0.05 are marked with an * and *p*-values below 0.01 are marked with ** for all figures. All the *p*-values were corrected using the Bonferroni–Holm approach.

Hemispherical preference was calculated through the laterality index for 4 Hz, Figure 8, and for 20 Hz, Figure 9. During 4 Hz stimulation, both groups presented right hemispherical preference across age. However, right lateralisation was stronger for the typically developing group at the age of 5 (*p* < 0.1) and 7 (*p* < 0.01). At the age of 9, both groups presented similar lateralisation values toward the right hemisphere. For 20 Hz stimulation, the preference for a right lateralisation was also present. The group with dyslexia had a stronger right lateralisation when compared to the weaker right hemispherical preference typical readers showed at the age of 5 (*p* < 0.1) and 7 (*p* < 0.01). However, at the age of 9, we could not find the differences between the two groups, despite both presented responses lateralised to the right.

## Discussion

### Source Localization

As it was stated in the results section, our figures for the source localization showed the averages (centre) and the jackknife estimates (error bars) for the dispersion around the mean. These error bars represent the distribution of the participants' source location for each spatial direction. Apart from the inherent limitation of the EEG regarding the spatial resolution, which is low compared to other techniques such as MRI, we have to remark upon the use of general MRI templates for all the ages. Due to the problems, we found that creating the BEM templates for our young participants, we decided to use an older functional BEM template based on the 14-year-old MRI. Since we used an older template, there are aspects that increase our limitations about spatial resolution: First, the size of the head and the distances between tissues are higher in adolescents than in children; another aspect is the geometrical distribution of these tissues, which differs from adolescents to children. In addition, using a general template for all the subjects, we are denying the presence of individual differences caused by a specific geometry of the head. The presented limitations cannot be quantified easily and be represented by an error bar. In summary, we showed these results as a qualitative measure of the nature of the responses. Based solely on the figures, we could not observe the differences between the processing of syllable-rate stimulation and phoneme-rate stimulation. The lack of difference was present along all the ages for both groups. From these results, we can conclude that with our methodology and our spatial resolution, the primary regions for speech processing in the brain are the same for syllable rate stimulation (4 Hz) as for phoneme-rate stimulation (20 Hz) in typically developing children as well as in children with dyslexia.

### Signal-to-Noise Ratio

#### Activity at 4 Hz (Theta Rhythm)

For accurate speech processing, the most important modulation frequencies are the ones below 16 Hz (Drullman et al., 1994a,b; Shannon et al., 1995). Moreover, modulation rates below 10 Hz have been associated in speech with the temporal rates of syllables (Greenberg et al., 2003; Edwards and Chang, 2013).

As it was shown in the Figure 4: at the age of 5, the children with dyslexia presented lower entrainment than the typically developing children; the same could be observed at the age of 9. This agrees with the hypotheses presented by Goswami (2011) where a lower entrainment was suggested in the theta (syllable) rate processing for the group with dyslexia. The age of 7 is an age when intensive reading training has already started. At this age, children with difficulties usually receive extra teaching support. The similarities in the level of neural entrainment found at this age might suggest that all the extra support received had its impact at a neurological level. In fact, this level of support intensifies in the later stages of reading development. Some intervention techniques for readers with dyslexia provide them with efficient tools to enhance their syllable perception. Sometimes, that comes at the cost of great mental effort that can be observed in a higher level of neural entrainment in adolescents and adults during stimulation with frequencies at syllable rates (Marosi et al., 1995; Arns et al., 2007; Lizarazu et al., 2015; De Vos et al., 2017b; Granados Barbero et al., 2021b). This extra effort is not always reflected at the neural amplitude level. However, we hypothesize that it has its impact by enhancing the SNR observed in children with dyslexia making it comparable to the typical readers (Hämäläinen et al., 2012; Poelmans et al., 2012).

#### Activity at 20 Hz (Beta Rhythm)

Modulation frequencies above 12 Hz are associated with the phoneme rate processing in speech.

As we showed in the results, before formal reading training (at 5 years of age), the group with dyslexia presented a higher level of entrainment. However, at the age of 9, the level of neural entrainment for this group is lower. We hypothesize that typically developing children will perform better after having received formal reading instruction for a long period of time and having trained their phonological representations, which reflected in the levels of neural entrainment. This may be increased by an atypical maturation of beta oscillations in dyslectic brains (De Vos et al., 2017b). This atypical maturation may explain the lack of differences in the levels of neural entrainment for both groups (Lehongre et al., 2011; Hämäläinen et al., 2012). Furthermore, it has been suggested that strong compensatory mechanisms would try to enhance the phoneme rate processing in people with dyslexia. These mechanisms might explain the higher levels of neural entrainment in adolescents with dyslexia (Granados Barbero et al., 2021b) or adults with dyslexia (Helenius et al., 2002) in comparison with typical readers.

### Connectivity

#### Activity at 4 Hz (Theta Rhythm)

At the age of 5, the level of connectivity is higher for the typical readers. This level of synchrony between the two main generators has its reflection as well in the level of neural entrainment represented by the SNR. During intensive reading training (7 years of age), the SNRs show no differences between the two groups. However, we can see that this might be a result of a higher neural effort represented in the higher levels of connectivity for the group with dyslexia. At the age of 9, both the SNR and the connectivity levels are higher for the typically developing children, suggesting a better neural processing of syllable-rate stimulation. The lower levels of neural entrainment and connectivity may reflect the effects of a disrupted neural network structure and mixed patterns of connectivity abnormalities in children with dyslexia (Frye et al., 2012; Koyama et al., 2013). Similar effects were found in resting-state experiments, where typically developing children presented higher levels of connectivity than the children with dyslexia in the theta frequency band (Fraga González et al., 2016; Xue et al., 2020). Strong compensatory mechanisms seem to appear in later stages of development. These mechanisms would cause the group with dyslexia to have higher levels of connectivity in adolescence (Granados Barbero et al., 2021b) and in adulthood (Thiede et al., 2020).

#### Activity at 20 Hz (Beta Rhythm)

The suggestion of an atypical maturation of beta oscillations in dyslexia (De Vos et al., 2017b) may explain the different levels of connectivity that can be found at the ages of 7 and 9. Typical readers show a higher phase coherence at the age of 7 while children with dyslexia have a higher phase coherence at the age of 9. Higher levels of connectivity in beta oscillations for children older than 8 years with dyslexia have also been reported in resting-state studies (Xue et al., 2020) and are present in adolescence (Granados Barbero et al., 2021b) and in adulthood (Thiede et al., 2020). This later maturation of beta oscillations found with connectivity may trigger the compensatory mechanisms in the neural entrainment levels (SNRs) that appear in adolescents (Granados Barbero et al., 2021b) and adults (Helenius et al., 2002).

### Laterality

It has been suggested that the right hemisphere is important for syllabic rate modulations (Poeppel, 2003; Boemio et al., 2005). We could indeed find during stimulation at 4 Hz a strong preference for right lateralisation even at the youngest age. The difference between groups that exists at the age of 5 and 7 might reflect a late maturation process for the children with dyslexia. Both groups would find a similar level of maturation at the age of 9, since both groups show a similar lateralisation level toward the right hemisphere. Different studies also found a right hemisphere lateralisation in theta rhythms in children (Abrams et al., 2008; Vanvooren et al., 2015) and in adults (Boemio et al., 2005; Bidelman and Howell, 2016).

For phonemic rate stimulation (~20 Hz), the preference for an ipsilateral lateralisation was clearly shown by the results, except at the age of 7 for the typically developing children. Both groups present similar levels of right hemispherical lateralisation at the age of 9. Before this age, the group with dyslexia shows a consistent preference for right lateralisation that is matched by the typically developing group at the age of nine. The preference for a right hemispherical lateralisation in both groups reached at the age of 9 would carry on toward adolescence as well (Granados Barbero et al., 2021b). This ipsilateral preference in phoneme-rate stimulation has been shown previously in adolescents and adults (Jamison et al., 2006; Obleser et al., 2008; Granados Barbero et al., 2021b).

## Conclusion

Despite the well-known heterogeneity during developmental stages, especially in children and in dyslexia, we could extract meaningful common oscillatory patterns using our methodology. In this longitudinal study, we confirmed that the existence of atypical levels of neural entrainment and connectivity already exists in pre-reading stages. Overall, these measures reflected a lower capability of the dyslectic brain to synchronize with syllable-rate stimulation, in the form of neural entrainment (SNR) and connectivity (phase coherence). In addition, our findings reinforced the hypothesis of a later maturation of the processing of beta rhythms in dyslexia. This study shows the importance of longitudinal studies in dyslexia, especially in children, where differences between the two groups can change in the span of a year. To conclude, we hope that we have provided important insights on syllabic and phonemic processing to understand the underlying differences in speech processing in developing children with dyslexia.

## Data Availability Statement

The data that support the findings of this study are available on request from the corresponding author, RGB. The data are not publicly available due to their containing information that could compromise the privacy of research participants.

## Ethics Statement

This study was approved by the Medical Ethical Committee of the University Hospitals of Leuven. Written informed consent to participate in this study was provided by the participants' legal guardian/next of kin.

## Author Contributions

RG and JW conceived of the presented idea. RG performed the implementation and the analytical computations. JW and PG supervised the project and the global findings of this work. RG and JW wrote the manuscript with input from PG. All authors contributed to the article and approved the submitted version.

## Funding

This study was supported by the European Union H2020 Marie Skłodowska-Curie Actions (MSCA)-ITN-2014-ETN Programme. Under the project advancing brain research in children's developmental neurocognitive disorders (ChildBrain, 641652). This work was partly supported by the Research Council of KU Leuven (C14/17/046) through a research grant to JW. Some of the resources and services used in this work were provided by the VSC (Flemish Supercomputer Centre), funded by the Research Foundation-Flanders (FWO) and the Flemish Government.

## Conflict of Interest

The authors declare that the research was conducted in the absence of any commercial or financial relationships that could be construed as a potential conflict of interest.

## Publisher's Note

All claims expressed in this article are solely those of the authors and do not necessarily represent those of their affiliated organizations, or those of the publisher, the editors and the reviewers. Any product that may be evaluated in this article, or claim that may be made by its manufacturer, is not guaranteed or endorsed by the publisher.

## References

[B1] Abrams D. A. Nicol T. Zecker S. Kraus N. (2008). Right-hemisphere auditory cortex is dominant for coding syllable patterns in speech. J. Neurosci. 28, 3958–3965. 10.1523/JNEUROSCI.0187-08.2008 18400895 PMC2713056

[B2] Arns M. Peters S. Breteler R. Verhoeven L. (2007). Different brain activation patterns in dyslexic children: evidence from EEG power and coherence patterns for the double-deficit theory of dyslexia. J. Integr. Neurosci. 06, 175–190. 10.1142/S0219635207001404 17472228

[B3] Bell A. J. Sejnowski T. J. (1995). An information-maximization approach to blind separation and blind deconvolution. Neural Comput. 7, 1129–1159. 10.1162/neco.1995.7.6.1129 7584893

[B4] Bidelman G. M. Howell M. (2016). Functional changes in inter- and intra-hemispheric cortical processing underlying degraded speech perception. Neuroimage 124, 581–590. 10.1016/j.neuroimage.2015.09.020 26386346

[B5] Boemio A. Fromm S. Braun A. Poeppel D. (2005). Hierarchical and asymmetric temporal sensitivity in human auditory cortices. Nat. Neurosci. 8, 389–395. 10.1038/nn1409 15723061

[B6] Brus B. T. Voeten M. J. M. (1973). Een-minuut-test: vorm A en B; Schoolvorderingentest voor de technische leesvaardigheid, bestemd voor het tweede tot en met het zesde leerjaar van het basisonderwijs; verantwoording en handleiding. Berkhout.

[B7] Cummins F. (2009). Rhythm as entrainment: the case of synchronous speech. J. Phon. 37, 16–28. 10.1016/j.wocn.2008.08.003

[B8] de Cheveigne A. Simon J. Z. (2008). Denoising based on spatial filtering. J. Neurosci. Methods 171, 331–339. 10.1016/j.jneumeth.2008.03.015 18471892 PMC2483698

[B9] De Vos A. Vanvooren S. Vanderauwera J. Ghesquière P. Wouters J. (2017a). A longitudinal study investigating neural processing of speech envelope modulation rates in children with (a family risk for) dyslexia. Cortex 93, 206–219. 10.1016/j.cortex.2017.05.007 28686908

[B10] De Vos A. Vanvooren S. Vanderauwera J. Ghesquière P. Wouters J. (2017b). Atypical neural synchronization to speech envelope modulations in dyslexia. Brain Lang. 164, 106–117. 10.1016/j.bandl.2016.10.002 27833037

[B11] Delorme A. Makeig S. (2004). EEGLAB: an open source toolbox for analysis of single-trial EEG dynamics including independent component analysis. J. Neurosci. Methods 134, 9–21. 10.1016/j.jneumeth.2003.10.009 15102499

[B12] Drullman R. Festen J. M. Plomp R. (1994a). Effect of reducing slow temporal modulations on speech reception. J. Acoust. Soc. Am. 95, 2670–2680. 10.1121/1.409836 8207140

[B13] Drullman R. Festen J. M. Plomp R. (1994b). Effect of temporal envelope smearing on speech reception. J. Acoust. Soc. Am. 95, 1053–1064. 10.1121/1.408467 8132899

[B14] Dudal P. (1997). Leerlingvolgsysteem VCLB (CSBO), Spelling: Toetsen 1-2-3. Basisboek en kopieerbundel [Student trajectory system. Spelling Grade 1-2-3. Manual]. Garant: Leuven.

[B15] Eden G. F. Olulade O. A. Evans T. M. Krafnick A. J. Alkire D. R. (2016). Chapter 65-developmental dyslexia, in Neurobiology of Language, eds Hickok G. Small S. L. (San Diego, CA: Academic Press), 815–826.

[B16] Edwards E. Chang E. F. (2013). Syllabic (~2–5 Hz) and fluctuation (~1–10 Hz) ranges in speech and auditory processing. Hear. Res. 305, 113–134. 10.1016/j.heares.2013.08.017 24035819 PMC3830943

[B17] Efron B. Stein C. (1981). The jackknife estimate of variance. Ann. Stat. 9, 586–596. 10.1214/aos/1176345462

[B18] Fraga González G. Van der Molen M. Žarić G. Bonte M. Tijms J. Blomert L.. (2016). Graph analysis of EEG resting state functional networks in dyslexic readers. Clin. Neurophysiol. 127, 3165–3175. 10.1016/j.clinph.2016.06.023 27476025

[B19] Frye R. E. Liederman J. McGraw Fisher J. Wu M.-H. (2012). Laterality of Temporoparietal causal connectivity during the prestimulus period correlates with phonological decoding task performance in dyslexic and typical readers. Cereb. Cortex 22, 1923–1934. 10.1093/cercor/bhr265 21980019 PMC3394369

[B20] Gabriel S. Lau R. W. Gabriel C. (1996). The dielectric properties of biological tissues: II. Measurements in the frequency range 10 Hz to 20 GHz. Phys. Med. Biol. 41, 2251–2269. 10.1088/0031-9155/41/11/002 8938025

[B21] Goossens T. Vercammen C. Wouters J. van Wieringen A. (2016). Aging affects neural synchronization to speech-related acoustic modulations. Front. Aging Neurosci. 8, 133. 10.3389/fnagi.2016.00133 27378906 PMC4908923

[B22] Goswami U. (2011). A temporal sampling framework for developmental dyslexia. Trends Cogn. Sci. 15, 3–10. 10.1016/j.tics.2010.10.001 21093350

[B23] Goswami U. (2015). Sensory theories of developmental dyslexia: three challenges for research. Nat. Rev. Neurosci. 16, 43–54. 10.1038/nrn3836 25370786

[B24] Granados Barbero R. De Vos A. Wouters J. (2021a). The identification of predominant auditory steady-state response brain sources in electroencephalography using denoising source separation. Eur. J. Neurosci. 53, 3688–3709. 10.1111/ejn.15219 33811405

[B25] Granados Barbero R. Vos A. Ghesquière P. Wouters J. (2021b). Atypical processing in neural source analysis of speech envelope modulations in adolescents with dyslexia. Eur. J. Neurosci. 54, 7839–7859. 10.1111/EJN.15515/v2/response1 34730259

[B26] Greenberg S. Carvey H. Hitchcock L. Chang S. (2003). Temporal properties of spontaneous speech—a syllable-centric perspective. J. Phon. 31, 465–485. 10.1016/j.wocn.2003.09.005

[B27] Guttorm T. K. Leppänen P. H. Poikkeus A.-M. Eklund K. M. Lyytinen P. Lyytinen H. (2005). Brain event-related potentials (ERPs) measured at birth predict later language development in children with and without familial risk for dyslexia. Cortex 41, 291–303. 10.1016/S0010-9452(08)70267-3 15871595

[B28] Hämäläinen J. A. Rupp A. Soltész F. Szücs D. Goswami U. (2012). Reduced phase locking to slow amplitude modulation in adults with dyslexia: an MEG study. Neuroimage 59, 2952–2961. 10.1016/j.neuroimage.2011.09.075 22001790

[B29] Hämäläinen M. S. Sarvas J. (1989). Realistic conductivity geometry model of the human head for interpretation of neuromagnetic data. IEEE Trans. Biomed. Eng. 36, 165–171. 10.1109/10.16463 2917762

[B30] Hebart M. N. Baker C. I. (2018). Deconstructing multivariate decoding for the study of brain function. Neuroimage 180, 4–18. 10.1016/j.neuroimage.2017.08.005 28782682 PMC5797513

[B31] Helenius P. Salmelin R. Richardson U. Leinonen S. Lyytinen H. (2002). Abnormal auditory cortical activation in dyslexia 100 msec after speech onset. J. Cogn. Neurosci. 14, 603–617. 10.1162/08989290260045846 12126501

[B32] Hofmann M. Wouters J. (2012). Improved electrically evoked auditory steady-state response thresholds in humans. J. Assoc. Res. Otolaryngol. 13, 573–589. 10.1007/s10162-012-0321-8 22569837 PMC3387309

[B33] Holm S. (1979). Board of the foundation of the scandinavian journal of statistics a simple sequentially rejective multiple test procedure a simple sequentially rejective multiple test procedure. Source 6, 65–70.

[B34] Hotelling H. (1931). The generalization of student's ratio. Ann. Math. Stat. 2, 360–378. 10.1214/aoms/1177732979

[B35] Jamison H. L. Watkins K. E. Bishop D. V. Matthews P. M. (2006). Hemispheric specialization for processing auditory nonspeech stimuli. Cereb. Cortex 16, 1266–1275. 10.1093/cercor/bhj068 16280465

[B36] Koyama M. S. Di Martino A. Kelly C. Jutagir D. R. Sunshine J. Schwartz S. J.. (2013). Cortical signatures of dyslexia and remediation: an intrinsic functional connectivity approach. PLoS ONE 8, e55454. 10.1371/journal.pone.0055454 23408984 PMC3569450

[B37] Lehongre K. Morillon B. Giraud A.-L. Ramus F. (2013). Impaired auditory sampling in dyslexia: further evidence from combined fMRI and EEG. Front. Hum. Neurosci. 7, 454. 10.3389/fnhum.2013.00454 23950742 PMC3738857

[B38] Lehongre K. Ramus F. Villiermet N. Schwartz D. Giraud A.-L. (2011). Altered low-gamma sampling in auditory cortex accounts for the three main facets of dyslexia. Neuron 72, 1080–1090. 10.1016/j.neuron.2011.11.002 22196341

[B39] Lins O. G. Picton T. W. (1995). Auditory steady-state responses to multiple simultaneous stimuli. Electroencephalogr. Clin. Neurophysiol. 96, 420–432. 10.1016/0168-5597(95)00048-W 7555916

[B40] Lizarazu M. Lallier M. Molinaro N. Bourguignon M. Paz-Alonso P. M. Lerma-Usabiaga G.. (2015). Developmental evaluation of atypical auditory sampling in dyslexia: Functional and structural evidence. Hum. Brain Mapp. 36, 4986–5002. 10.1002/hbm.22986 26356682 PMC6869042

[B41] Marosi E. Harmony T. Becker J. Reyes A. Bernal J. Fern'andez T.. (1995). Electroencephalographic coherences discriminate between children with different pedagogical evaluation. Int. J. Psychophysiol. 19, 23–32. 10.1016/0167-8760(94)00059-N 7790286

[B42] McAnally K. I. Stein J. F. (1997). Scalp potentials evoked by amplitude-modulated tones in dyslexia. J. Speech Lang. Hear. Res. 40, 939–945. 10.1044/jslhr.4004.939 9263956

[B43] Meijs J. W. H. Weier O. W. Peters M. J. Oosterom A. V. (1989). On the numerical accuracy of the boundary element method (eeg application). IEEE Trans. Biomed. Eng. 36, 1038–1049. 10.1109/10.40805 2793196

[B44] Menell P. McAnally K. I. Stein J. F. (1999). Psychophysical sensitivity and physiological response to amplitude modulation in adult dyslexic listeners. J. Speech Lang. Hear. Res. 42, 797–803. 10.1044/jslhr.4204.797 10450901

[B45] Miller R. G. (1974). The jackknife-a review. Biometrika 61, 1–15. 10.1093/biomet/61.1.1

[B46] Obleser J. Eisner F. Kotz S. A. (2008). Bilateral speech comprehension reflects differential sensitivity to spectral and temporal features. J. Neurosci. 28, 8116–8123. 10.1523/JNEUROSCI.1290-08.2008 18685036 PMC6670773

[B47] Onton J. (2009). High-frequency broadband modulation of electroencephalographic spectra. Front. Hum. Neurosci. 3, 2009. 10.3389/neuro.09.061.2009 20076775 PMC2806183

[B48] Onton J. Westerfield M. Townsend J. Makeig S. (2006). Imaging human EEG dynamics using independent component analysis. Neurosci. Biobehav. Rev. 30, 808–822. 10.1016/j.neubiorev.2006.06.007 16904745

[B49] Oostenveld R. Fries P. Maris E. Schoffelen J. M. (2011). FieldTrip: open source software for advanced analysis of MEG, EEG, and invasive electrophysiological data. Comput. Intell. Neurosci. 2011. 10.1155/2011/156869 21253357 PMC3021840

[B50] Picton T. W. Dimitrijevic A. Sasha John M. Van Roon P. (2001). The use of phase in the detection of auditory steady-state responses. Clin. Neurophysiol. 112, 1698–1711. 10.1016/S1388-2457(01)00608-3 11514253

[B51] Poelmans H. Luts H. Vandermosten M. Ghesquière P. Wouters J. (2012). Hemispheric asymmetry of auditory steady-state responses to monaural and diotic stimulation. J. Assoc. Res. Otolaryngol. 13, 867–876. 10.1007/s10162-012-0348-x 22926721 PMC3505592

[B52] Poeppel D. (2003). The analysis of speech in different temporal integration windows: cerebral lateralization as ‘asymmetric sampling in time’. Speech Commun. 41, 245–255. 10.1016/S0167-6393(02)00107-3

[B53] Rance G. (2008). The Auditory Steady-state Response: Generation, Recording, and Clinical Application. San Diego, CA: Plural Publishing.

[B54] Raven J. Court J. (1996). Manual for Raven's Progressive Matrices and Vocabulary Scales: Standard progressive matrices. Manual for Raven's Progressive Matrices and Vocabulary Scales. Oxford: Oxford Psychologists Press.

[B55] Richards J. E. Sanchez C. Phillips-Meek M. Xie W. (2016). A database of age-appropriate average MRI templates. Neuroimage 124, 1254–1259. 10.1016/j.neuroimage.2015.04.055 25941089 PMC4630162

[B56] Sanchez C. E. Richards J. E. Almli C. R. (2012). Age-Specific MRI Templates for Pediatric Neuroimaging. Dev. Neuropsychol. 37, 379–399. 10.1080/87565641.2012.688900 22799759 PMC3399736

[B57] Särelä J. Valpola H. (2005). Denoising source separation. J. Mach. Learn. Res. 6, 233–272.

[B58] Shannon R. V. Zeng F.-G. Kamath V. Wygonski J. Ekelid M. (1995). Speech recognition with primarily temporal cues. Science 270, 303–304. 10.1126/science.270.5234.303 7569981

[B59] Share D. L. (2021). Common misconceptions about the phonological deficit theory of dyslexia. Brain Sci. 11, 1510. 10.3390/brainsci11111510 34827508 PMC8615585

[B60] Stoodley C. J. (2016). Chapter 9-the role of the cerebellum in developmental dyslexia, in The Linguistic Cerebellum, eds Mariën P. M. Manto (San Diego, CA: Academic Press), 199–221.

[B61] Thiede A. Glerean E. Kujala T. Parkkonen L. (2020). Atypical MEG inter-subject correlation during listening to continuous natural speech in dyslexia. Neuroimage 216, 116799. 10.1016/j.neuroimage.2020.116799 32294536

[B62] Van den Bos K. Spelberg H. Scheepsma A. De Vries J. (1994). De klepel. vorm a en b. een test voor de leesvaardigheid van pseudowoorden. verantwoording, handleiding, diagnostiek en behandeling. Nijmegen: Berkhout.

[B63] Van der Reijden C. S. Mens L. H. M. Snik A. F. M. (2005). EEG derivations providing auditory steady-state responses with high signal-to-noise ratios in infants. Ear Hear. 26, 299–309. 10.1097/00003446-200506000-00006 15937411

[B64] Van Dun B. Wouters J. Moonen M. (2009). Optimal electrode selection for multi-channel electroencephalogram based detection of auditory steady-state responses. J. Acoust. Soc. Am. 126, 254–268. 10.1121/1.3133872 19603882

[B65] Van Wieringen A. Wouters J. (2008). LIST and LINT: sentences and numbers for quantifying speech understanding in severely impaired listeners for Flanders and the Netherlands. Int. J. Audiol. 47, 348–355. 10.1080/14992020801895144 18569107

[B66] Vandermosten M. Correia J. Vanderauwera J. Wouters J. Ghesqui‘ere P. Bonte M. (2020). Brain activity patterns of phonemic representations are atypical in beginning readers with family risk for dyslexia. Dev. Sci. 23, 1–15. 10.1111/desc.12857 31090993

[B67] Vanvooren S. Hofmann M. Poelmans H. Ghesquière P. Wouters J. (2015). Theta, beta and gamma rate modulations in the developing auditory system. Hear. Res. 327, 153–162. 10.1016/j.heares.2015.06.011 26117409

[B68] Vanvooren S. Poelmans H. Hofmann M. Ghesquière P. Wouters J. (2014). Hemispheric asymmetry in auditory processing of speech envelope modulations in prereading children. J. Neurosci. 34, 1523–1529. 10.1523/JNEUROSCI.3209-13.2014 24453339 PMC6705306

[B69] Vellutino F. R. Fletcher J. M. Snowling M. J. Scanlon D. M. (2004). Specific reading disability (dyslexia): what have we learned in the past four decades? J. Child Psychol. Psychiatry 45, 2–40. 10.1046/j.0021-9630.2003.00305.x 14959801

[B70] Xue H. Wang Z. Tan Y. Yang H. Fu W. Xue L.. (2020). Resting-state EEG reveals global network deficiency in dyslexic children. Neuropsychologia 138, 107343. 10.1016/j.neuropsychologia.2020.107343 31952981

[B71] Ziegler J. C. Goswami U. (2005). Reading Acquisition, developmental dyslexia, and skilled reading across languages: a psycholinguistic grain size theory. Psychol. Bull. 131, 3–29. 10.1037/0033-2909.131.1.3 15631549

